# Benefits of Macitentan in Patients with Pulmonary Hypertension: A Systematic Review and Meta-Analysis of Randomized Controlled Trials

**DOI:** 10.5334/gh.1274

**Published:** 2023-10-26

**Authors:** Jinlv Qin, Guizuo Wang, Dong Han

**Affiliations:** 1 Radioimmunoassay Center, Shaanxi Provincial People’s Hospital, Xi’an, Shaanxi 710068, China; 2 Department of Respiratory and Critical Care Medicine, Shaanxi Provincial People’s Hospital, Xi’an, Shaanxi 710068, China

**Keywords:** macitentan, pulmonary hypertension, efficacy, meta-analysis

## Abstract

**Background:**

This systematic review and meta-analysis aimed to determine the efficacy of macitentan in patients with pulmonary hypertension (PH).

**Methods:**

A systematic search was made of PubMed, Embase, Cochrane Library, and clinicaltrials.gov, without language restrictions. Randomized controlled trials (RCTs) on treatment of PH with macitentan, compared with placebo or blank, were reviewed. Studies were pooled to weighted mean differences (WMDs) and risk ratios (RRs), with 95% confidence intervals (CIs).

**Results:**

Six RCTs (enrolling 1,003 participants) met the inclusion criteria. Macitentan showed significant effects on 6-min walk distance (6MWD) (WMD 12.06 m, 95% CI 2.12 to 21.99 m), pulmonary vascular resistance (PVR) (WMD –186.51 dyn·s/cm^–5^, 95% CI –232.72 to –140.29 dyn·s/cm^–5^), mean pulmonary artery pressure (mPAP) (WMD –3.20 mmHg, 95% CI –5.93 to –0.47 mmHg), N-terminal pro-brain natriuretic peptide (NT-proBNP) (WMD –232.47 ng/L, 95% wCI –318.22 to –146.72 ng/L), and cardiac index (WMD 0.39 L/min/m^2^, 95% CI 0.20 to 0.58 L/min/m^2^).

**Conclusion:**

Macitentan significantly improved 6MWD, PVR, mPAP, NT-proBNP, and cardiac index in patients with PH. Macitentan should be further validated in patients with PH.

## Introduction

Pulmonary hypertension (PH) is a life-threatening disease defined as mean pulmonary arterial pressure (mPAP) ≥20 mmHg at rest, as measured by right heart catheterization [1,2]. According to the mechanism or underlying etiology, PH is divided into five clinical groups by the World Health Organization (WHO): Pulmonary arterial hypertension (PAH), PH secondary to left heart disease (PH-LHD), PH associated with hypoxemia, PH due to chronic thrombotic disease, embolic disease, or both, and miscellaneous [3,4]. Despite the availability of treatments for all subgroups of PH, the prognosis for PH remains poor [3,5]. The dual endothelin receptor antagonist (ERA) macitentan is characterized by sustained receptor binding [6], which is achieved by modifying the structure of bosentan to improve efficacy and safety [7]. Only a few randomized controlled trials (RCTs) have evaluated the efficacy of macitentan in patients with PH, and their results differed.

Therefore, the aim of this study was to perform a systematic review and meta-analysis of RCTs to determine the efficacy of macitentan in patients with PH.

## Methods

### Data sources and search strategy

This systematic review and meta-analysis was based on the Preferred Reporting Items for Systematic Reviews and Meta-Analyses (PRISMA) statement [8]. The protocol was previously registered in November 2022 in the PROSPERO database (Review register: CRD42022371410). The PubMed, Embase, Cochrane Library, and clinicaltrials.gov were searched for studies up to November 2022.

### Study selection

To be eligible for inclusion in the meta-analysis studies had to meet the following criteria: (a) inclusion of patients with PH aged ≥12 years, mPAP ≥25 mmHg, pulmonary vascular resistance (PVR) ≥240 dyn·s/cm^–5^, and 6-min walk distance (6MWD) ≥50 m, according to definition of WHO [1]; (b) use of a randomized controlled design to make a comparison of macitentan (10 mg once daily) with placebo or blank; and (c) follow-up for 12 weeks or longer to observe the efficacy. Studies were excluded if they included patients with the following: (a) severe co-existing diseases with poor functional status (eg, advanced renal failure, severe hepatic impairment); (b) current ERAs treatment; (c) previous pulmonary endarterectomy (PEA) or balloon pulmonary angioplasty (BPA); (d) pregnant or lactating women; and (e) drug or alcohol abuse. The search strings used for the databases were (‘macitentan’ OR ‘Actelion-1’ OR ‘ACT-064992’) AND (‘pulmonary arterial hypertension’ OR ‘pulmonary hypertension’ OR ‘pulmonary artery pressure’ OR ‘PH’). The reference lists of any relevant review articles were also screened to identify studies that might have been missed in this search. No language restrictions were applied to our study selection process.

### Data extraction and quality assessment

Two reviewers independently screened articles according to the inclusion criteria. The reviewers compared selected studies and differences were resolved by consensus. A third reviewer acted as arbitrator in case of discrepancy between reviewers. Data tables were used to collect all relevant data from texts, tables and figures of each included trial, including author, year of publication or last update posted, patient number and age, duration of follow-up, treatment category, baseline 6MWD, WHO functional class (FC), and outcomes such as 6MWD, PVR, mPAP, N-terminal pro-brain natriuretic peptide (NT-proBNP), cardiac index, improvement in WHO FC, hospitalization for worsening of PH, death due to PH, and all-cause death. Study quality was assessed using the Detsky Quality Assessment Scale [9,10,11,12,13]. This is a 20-point scale for studies with statistically significant results and a 21-point scale for studies without statistically significant results.

### Risk of bias of included trials

Two reviewers independently assessed the risk of bias using the Cochrane collaboration risk of bias tool for RCTs [14].

### Data synthesis and statistical analysis

Meta-analyses were conducted where applicable; otherwise, outcomes were presented in narrative form. Data were analyzed using the RevMan Version 5.4.1 (The Cochrane Collaboration). Next, risk ratios (RRs) for discontinuous outcomes, and weighted mean differences (WMDs) for continuous outcomes, with corresponding 95% confidence intervals (CIs) were computed for individual trials. Chi-squared and Higgins I2 tests were used to assess heterogeneity among included trials. If significant heterogeneity (p ≤ 0.10 for Chi-squared test results or I2 ≥ 50%) was obtained, we used a random-effects model, otherwise a fixed-effects model was used. And a P value <0.05 was taken to indicate statistical significance. The P value of Egger’s linear regression test (STATA version 12.0) was used to assess the presence of publication bias in included studies for each outcome.

## Results

### Study selection and characteristics

**Figure 1:**
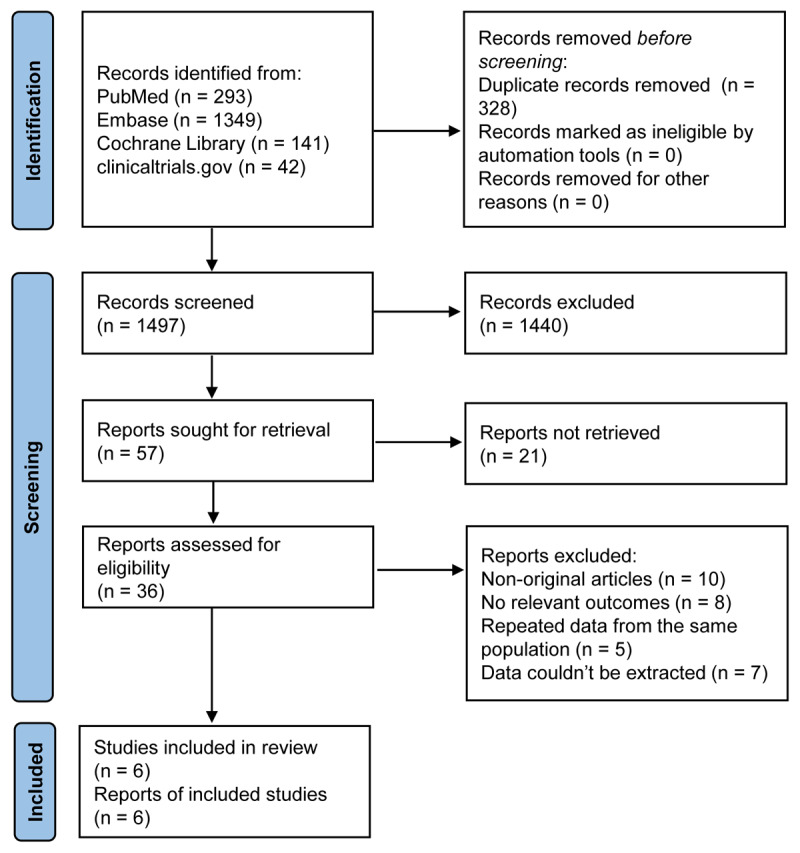
Flow chart for selection of studies.

Of 1,825 studies recognized by the initial search, 57 were retrieved for more detailed assessment, and 6 trials [15,16,17,18,19,20,28] were included in this meta-analysis (Figure 1). Baseline characteristics of trials included in this meta-analysis are shown in Table 1. A total of 1,003 patients were included: 498 assigned to the macitentan treatment groups and 505 to the control groups. The risk of bias results are summarized in Figure S1.

**Table 1:** Baseline characteristics of trials included in meta-analysis.


STUDY(REF. #)	YEAR	QUALITY SCORE	FOLLOW-UP WEEKS	PH TYPE	REGIMEN	n	AGE, YEARS (SD)	MALE, %	6MWD, M (SD)	WHO FC, %			

										I	II	III	IV

Gatzoulis (15)	2019	19	16	PAH	Macitentan	114	33 *	28.1	368.7 (74.5)	0	60.5	30.5	0

					Placebo	112	31 *	39.3	380.3 (76.3)	0	58.9	41.1	0

Ghofrani (16)	2017	20	24	CTEPH	Macitentan	40	58.2 (14.0)	35	353.0 (87.9)	0	30	70	0

					Placebo	40	56.9 (13.9)	38	351.2 (73.8)	0	15	82.5	2.5

Pulido (17)	2013	18	104	PAH	Macitentan	242	44.5 (16.3)	19.8	363 (93.2)	0.4	49.6	47.9	2.1

					Placebo	250	46.7 (17.0)	26.1	352 (110.6)	0	51.8	46.6	1.6

Sitbon (19)	2019	21	12	PAH	Macitentan	43	58.0 (8.7)	51	385.8 (100.0)	2	63	35	0

					Placebo	42	59.0 (9.5)	52	383.2 (108.9)	2	55	43	0

Vachiéry (20)	2018	17	12	PH-LHD	Macitentan	31	70.0 *	19.4	300 *	0	16.1	83.9	0

					Placebo	32	72.0 *	50.0	305 *	0	31.3	68.8	0

SOPRANO (28)	2021	16	12	PH-LHD	Macitentan	28	56.5 (8.2)	78.6	NR	NR	NR	NR	NR

					Placebo	29	58.2 (7.0)	79.3	NR	NR	NR	NR	NR


**Abbreviations:** CTEPH, chronic thromboembolic pulmonary hypertension; 6MWD, 6-min walk distance; NR, not reported; PAH, pulmonary arterial hypertension; PH, pulmonary hypertension; PH-LHD, PH secondary to left heart disease; SD, standard deviation; WHO FC, World Health Organization functional class. * Median.

### 6-Min walk distance

**Figure 2:**
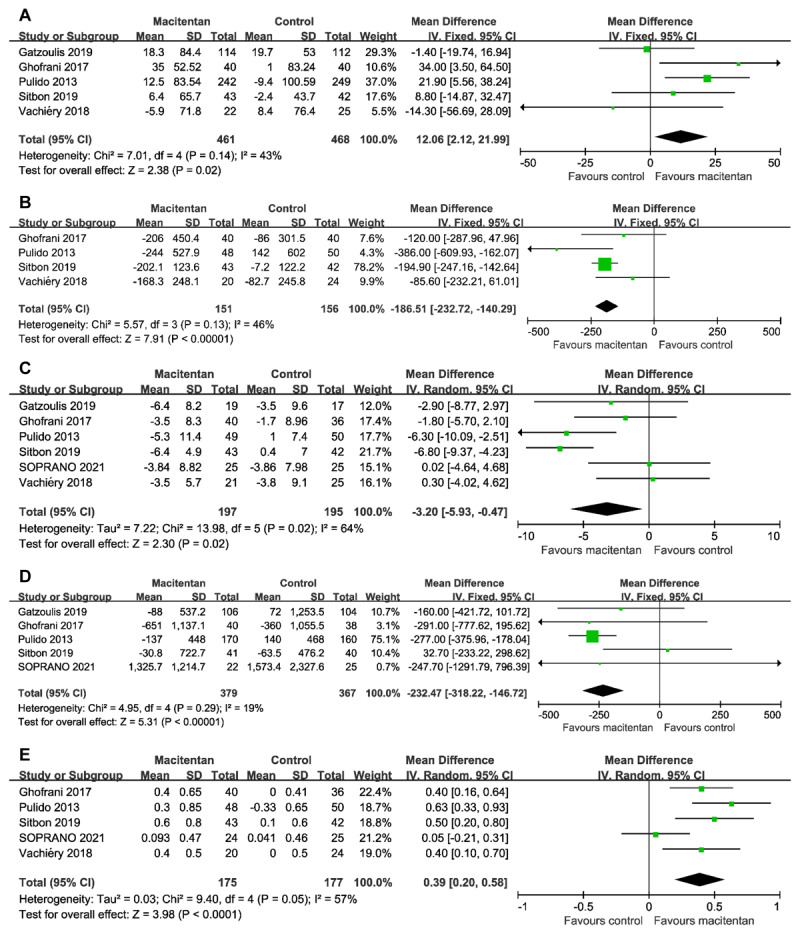
Forest plot assessing the efficacy of macitentan on **(A)** 6-min walk distance, **(B)** pulmonary vascular resistance, **(C)** mean pulmonary artery pressure, **(D)** NT-proBNP, and **(E)** cardiac index.

Data on the change of 6MWD were extracted from five RCTs (929 patients). Compared with the control conditions, macitentan treatment significantly improved 6MWD (WMD 12.06 m, 95% CI 2.12 to 21.99 m; P = 0.02 [Figure 2A]). There was no significant heterogeneity (I2 = 43%; P = 0.14). Egger’s test (P = 0.742) did not show evidence of publication bias.

### Pulmonary vascular resistance

The change of PVR was evaluated in four RCTs (307 patients). Compared with the control conditions, macitentan treatment significantly decreased PVR (WMD –186.51 dyn·s/cm^–5^, 95% CI –232.72 to –140.29 dyn·s/cm^–5^; P < 0.00001 [Figure 2B]). There was no significant heterogeneity (I2 = 46%; P = 0.13). Egger’s test (P = 0.980) did not show evidence of publication bias.

### Mean pulmonary artery pressure

The change of mPAP was evaluated in six randomized studies (392 patients). Compared with the control conditions, macitentan treatment significantly decreased mPAP (WMD –3.20 mmHg, 95% CI –5.93 to –0.47 mmHg; P = 0.02 [Figure 2C]). There was significant heterogeneity (I2 = 64%; P = 0.02). Egger’s test (P = 0.271) did not show evidence of publication bias.

### N-terminal pro-brain natriuretic peptide

Data on the change of NT-proBNP were extracted from five RCTs (746 patients). Compared with the control conditions, the use of macitentan significantly decreased the level of NT-proBNP (WMD –232.47 ng/L, 95% CI –318.22 to –146.72 ng/L; P < 0.00001 [Figure 2D]). There was no significant heterogeneity (I2 = 19%; P = 0.29). Egger’s test (P = 0.200) did not show evidence of publication bias.

### Cardiac index

Data on the change of cardiac index were available from five trials (352 patients). Compared with the control groups, macitentan significantly improved cardiac index (WMD 0.39 L/min/m^2^, 95% CI 0.20 to 0.58 L/min/m^2^; P < 0.0001 [Figure 2E]). There was significant heterogeneity (I2 = 57%; P = 0.05). Egger’s test (P = 0.414) did not show evidence of publication bias.

### Improvement in WHO functional class (FC)

**Figure 3:**
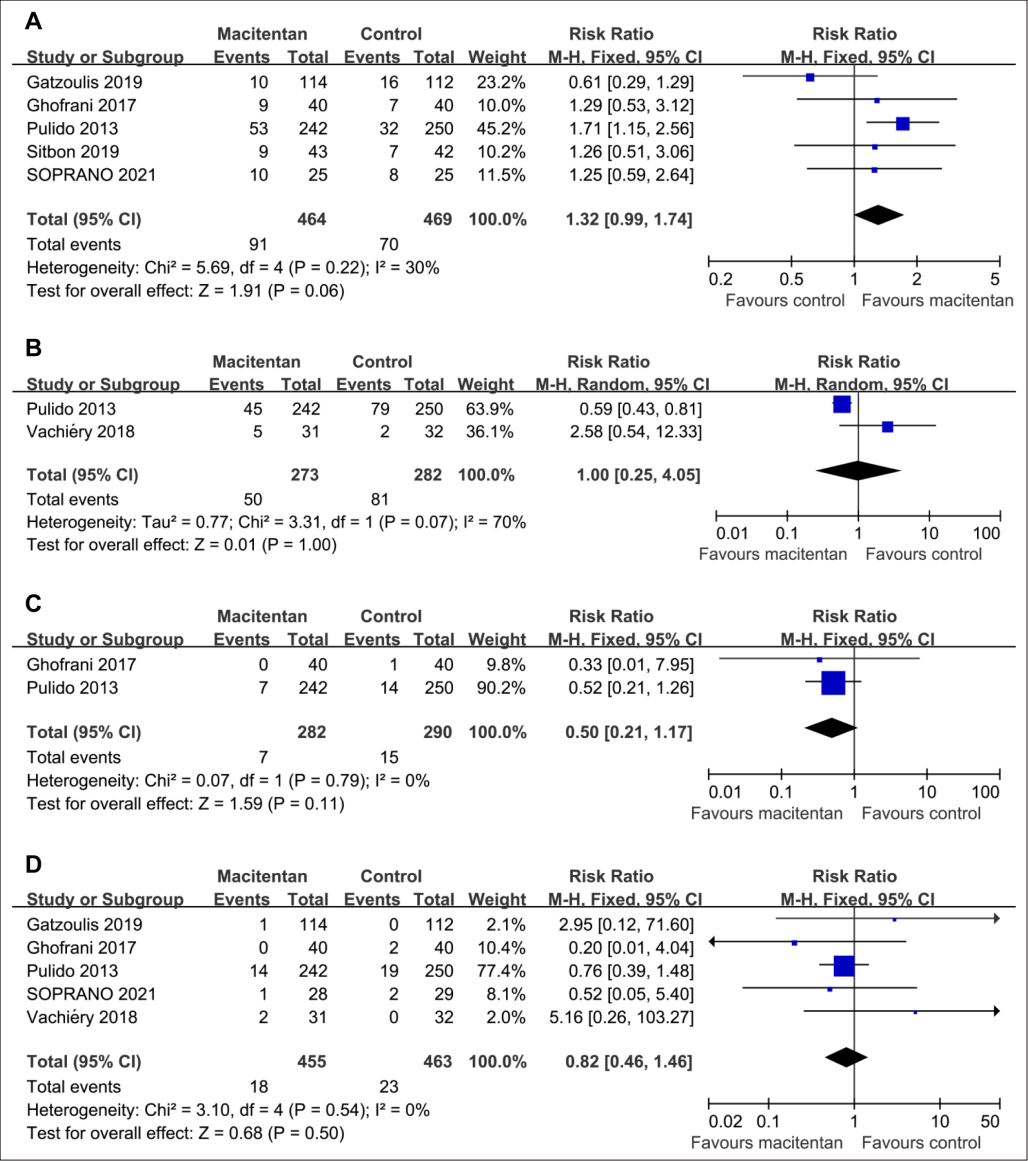
Forest plot assessing the efficacy of macitentan on **(A)** improvement in WHO FC, **(B)** hospitalization for worsening of PH, **(C)** PH-related death, and **(D)** all-cause death.

Five RCTs reported data on improvement in WHO FC (933 patients). The percentage of patients with improvement in WHO FC did not differ significantly between the two groups (RR 1.32, 95% CI 0.99 to 1.74; P = 0.06 [Figure 3A]). There was no significant heterogeneity (I2 = 30%; P = 0.22). And the percentage in macitentan groups was 19.61% compared with 14.93% in control groups. Egger’s test (P = 0.251) did not show evidence of publication bias.

### Hospitalization for worsening of PH

Two trials reported data on hospitalization for worsening of PH (including 555 patients). The percentage of patients with hospitalization for worsening of PH did not differ significantly between the two groups (RR 1.00, 95% CI 0.25 to 4.05; P = 1.00 [Figure 3B]). There was significant heterogeneity among the studies (I2 = 70%; P = 0.07). And the percentage in macitentan groups was 18.32% compared with 28.72% in control groups.

### P-Hrelated death

Five RCTs reported data on PH-related death (946 patients), and only two trials had patient deaths. There was no statistically significant difference in PH-related mortality between the two groups (RR 0.50, 95% CI 0.21 to 1.17; P = 0.11 [Figure 3C]). There was no significant heterogeneity (I2 = 0%; P = 0.79). The PH-related mortality in macitentan groups was 1.49% compared with 3.15% in control groups.

### All-cause death

Six RCTs reported data on all-cause death (1,003 patients), and five trials had patient deaths. There was no statistically significant difference in all-cause mortality between the two groups (RR 0.82, 95% CI 0.46 to 1.91; P = 0.50 [Figure 3D]). There was no significant heterogeneity (I2 = 0%; P = 0.54). The all-cause mortality in macitentan groups was 3.61% compared with 4.55% in control groups. Egger’s test (P = 0.678) did not show evidence of publication bias.

## Discussion

This systematic review and meta-analysis is designed specifically to evaluate RCTs that have explored the efficacy of macitentan in patients with PH. Based on the current results, we observed that macitentan significantly improved 6MWD, PVR, mPAP, NT-proBNP, and cardiac index. There was a trend for improvement in WHO FC and reduction in PH-related deaths with macitentan, although not statistically significant.

PH is a major global health problem. The prevalence of PH in the global population is about 1%, and even reaches 10% in people over 65 years old [21]. In recent years, research on macitentan has focused on PAH, PH-LHD, and chronic thromboembolic PH (CTEPH). Pulmonary arterial hypertension (group 1) is characterized by loss and obstructive remodeling of the pulmonary vascular bed. Pulmonary arterial hypertension is characterized by precapillary PH, defined as mPAP ≥20 mm Hg, pulmonary artery wedge pressure (PAWP) ≤15 mm Hg, and PVR ≥3 Wood units (WU) [1]. Chronic elevation of PVR may cause progressive right ventricular (RV) dysfunction and RV failure (RVF) [22]. Persistence of RVF may lead to increased right atrial pressure and decreased cardiac index [3]. Despite guidelines recommending combination therapy targeting multiple pathways of PAH, patient outcomes remain poor [21,23]. PH-LHD (group 2) is initially caused by a passive increase in left atrial pressure and develops further in response to endothelial dysfunction and vasoconstriction [24]. In patients with chronic heart failure and PH-LHD, elevated plasma endothelin-1 levels are associated with increased pulmonary pressure [25] and greater risk of death [26]. Chronic thromboembolic PH (group 4) is characterized by pulmonary artery obstruction and vascular remodeling caused by chronic organized thrombus. Although CTEPH has treatment options of PEA and BPA. Some patients who are ineligible for treatment or have residual or recurrent PH still lack effective treatments.

## Limitations

This study met most of the methodological criteria recommended for systematic reviews and meta-analyses [27]. However, some limitations need to be considered when interpreting the results of this study. Firstly, some included studies had small sample sizes, which may have reduced the power of the results. Secondly, the number of included studies was small. Thirdly, some potential confounding between-study variables could have influenced outcomes and thus this may have also affected our meta-analysis results. Fourthly, this meta-analysis included three different clinical groups of PH that may respond differently to macitentan. Finally, because the inability to analyze individual patient data, this pooled data meta-analysis was not patient-level, and there may be within-study heterogeneity as well as between-study heterogeneity, so the results should be considered provisional.

Future RCTs should elucidate the efficacy of macitentan on long-term outcomes of PH, particularly mortality, and explore the possibilities of macitentan in different clinical groups with PH.

## Conclusion

Macitentan significantly improved 6MWD, PVR, mPAP, NT-proBNP, and cardiac index in patients with PH. Macitentan should be further validated in patients with PH.

## Data Accessibility Statement

Extracted data are available on request to the corresponding author.

## Additional Files

The additional files for this article can be found as follows:

10.5334/gh.1274.s1 Supplementary File 1. Figure s1.

10.5334/gh.1274.s2 Supplementary File 2. PRISMA 2020 Checklist.
