# Supplementary figures and images for: Benefits of Macitentan in Patients with Pulmonary Hypertension: A Systematic Review and Meta-Analysis of Randomized Controlled Trials
Source: Glob Heart. 2023 Oct 26;18(1):58. doi: 10.5334/gh.1274 (PMC10607635; doi:10.5334/gh.1274)

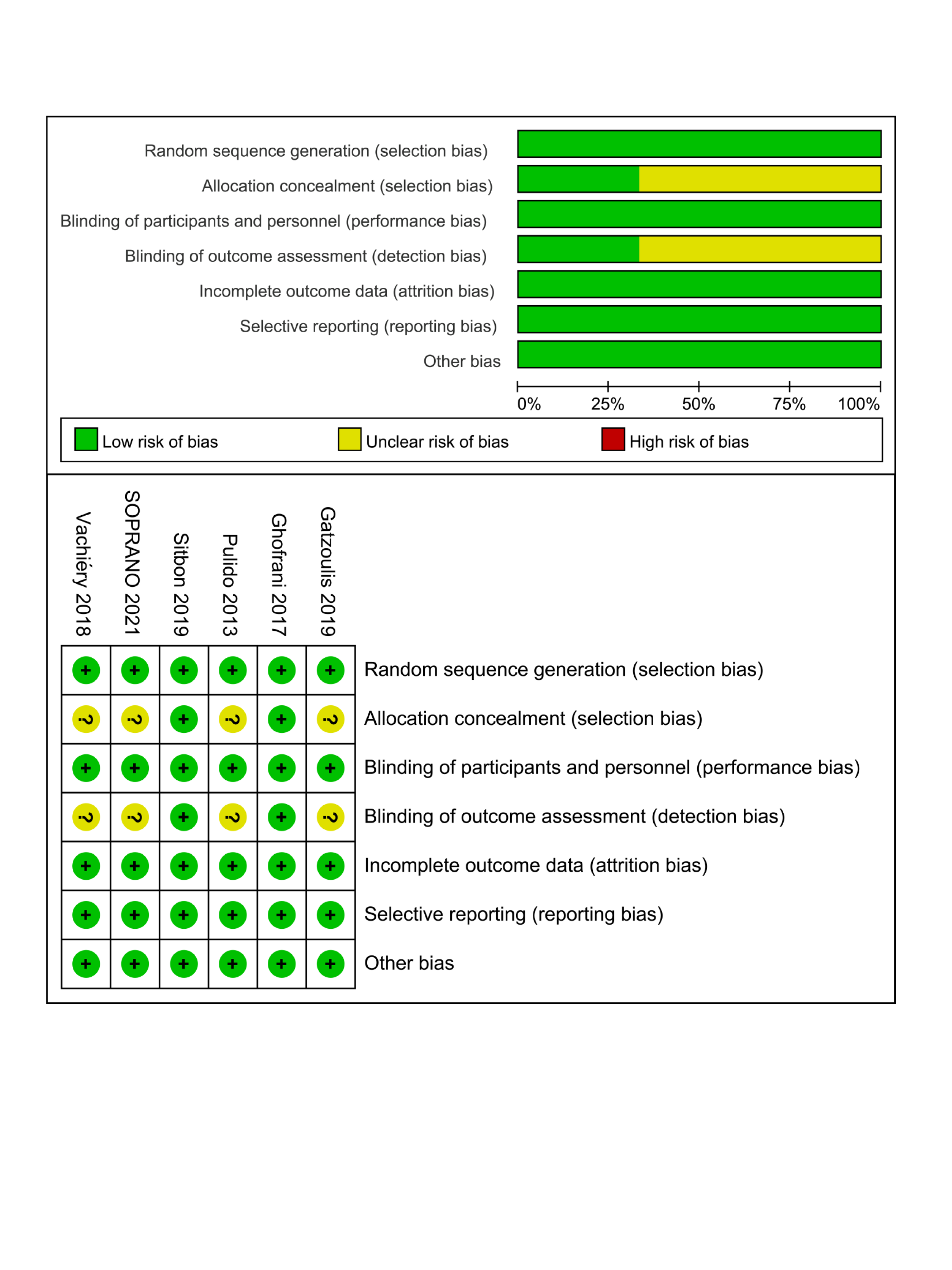

Supplement: Supplementary File 1. — Figure s1. [file gh-18-1-1274-s1.tiff]
